# Introducing video consultations at public sexual health clinics in the Netherlands: a mixed-methods study

**DOI:** 10.1093/heapro/daac135

**Published:** 2022-09-29

**Authors:** Filippo Zimbile, Silke David, Maud Daemen, Anne Goossens, Josien Creemers, Rik Crutzen

**Affiliations:** Department of Health Promotion, Maastricht University/CAPHRI, Maastricht, The Netherlands; National Institute for Public Health and the Environment (RIVM), Bilthoven, The Netherlands; Aidsfonds – Soa Aids Nederland, Amsterdam, The Netherlands; National Institute for Public Health and the Environment (RIVM), Bilthoven, The Netherlands; Department of Health Promotion, Maastricht University/CAPHRI, Maastricht, The Netherlands; Department of Health Promotion, Maastricht University/CAPHRI, Maastricht, The Netherlands; Department of Health Promotion, Maastricht University/CAPHRI, Maastricht, The Netherlands; Department of Health Promotion, Maastricht University/CAPHRI, Maastricht, The Netherlands

**Keywords:** video consultation, STI, sexual health, patient satisfaction, access

## Abstract

Video consultations (in combination with remote STI testing) can benefit both public sexual health clinics (SHCs) and their clients. The Dutch public SHCs explored the extent to which video consultations are accepted and appreciated—compared to face-to-face consultations—by both young clients (under 25 years) and nurses who normally carry out consultations. A mixed-methods study, using online questionnaires and telephone interviews with both young clients (aged under 25 years) and nurses (focus groups), was conducted to evaluate acceptance and appreciation of video and face-to-face consultations of the SHCs. Young clients evaluated 333 video consultations and 100 face-to-face consultations. Clients rated the VCs and F2F consultations as being of equal high level on five evaluation criteria (e.g. how it feels to talk about sex with a nurse, contact with the nurse). These positive results were confirmed in the interviews. Most important perceived advantages of VCs were time saving, ease, and feelings of comfort and safety. The nurses evaluated 422 VCs and 120 F2F consultations, rating the VCs and F2F consultations on an equal high level on three evaluation criteria (e.g., contact with the client, possibility to continue asking questions). Increasing accessibility of SHC consultations, getting faster to the point and saving time were mentioned as advantages of VCs during the focus group sessions with nurses. Video consultations are accepted and appreciated by young clients and nurses. They can be used for standard STI consultations that do not require a physical examination.

## INTRODUCTION

The 24 sexual health clinics (SHCs) at the municipal public health centres covering the Netherlands provide sexual-transmitted infections (STIs) testing for high-risk groups without a referral from a medical professional. These high-risk groups include individuals with STI-related symptoms, young people under 25 years of age, men having sex with men (MSM) and people originating from—or having a partner from—a country with a high prevalence of STIs and HIV. More than half (51%) of the annually 150 000 STI consultations at SHCs are provided to attendees under 25 (Staritsky *et al.*, 2020). In addition to STI consultations, young people can also anonymously contact SHCs free-of-charge for information and personal consultations on a broad range of subjects related to sexual health. Subjects include unwanted pregnancy, birth control, gender identity and sexual orientation. In 2019, about 11 000 sexuality consultations were held by SHCs (Staritsky *et al.*, 2020).

As in many other Western countries access to public sexual and reproductive health (SRH) services in the Netherlands are limited for young people due to barriers they experience with clinical visits (Bender and Fulbright, 2013; Gan *et al.*, 2021). A review study revealed that confidentiality and being able to visit a clinic without being seen are of great importance to young people (Bender and Fulbright, 2013). These barriers are especially relevant in smaller rural communities where lack of privacy plays a greater role. Other factors that are known to influence the accessibility of SRH services are health literacy, sexual health knowledge, knowledge of health systems, health seeking behaviour, affordability (e.g., travel costs to the clinic) and language barriers (Slater and Robinson, 2014; Berglas *et al.*, 2016; Wasserman *et al.*, 2019). At the same time, research in various countries shows that STIs are more prevalent among ethnic minorities and people with a low-socio-economic position and that these groups make less use of the services of the SHCs compared with the rest of the population (Matser *et al.*, 2013; Slater and Robinson, 2014; Oeffelen *et al.*, 2017; Coyle *et al.*, 2018; Ostendorf *et al.*, 2021). Public SHCs are therefore keen to strengthen the reach of their services among these target groups (Götz *et al.*, 2019).

Remote testing can be attractive for young people because they offer the possibility of avoiding travelling and reducing patients’ expenditure. Online services can prevent discomfort such as feelings of shame, that some clients experience with face-to-face (F2F) consultations for sexual health (Hottes *et al.*, 2012; Lorimer and McDaid, 2013; Minichiello *et al.*, 2013). By reducing privacy concerns and facilitating remote access to testing, young people might be more likely to test, or test more often (Aicken *et al.*, 2016). Remote testing has the potential to increase willingness to test among those most in need (Wilson *et al.*, 2017).

Besides the potential benefit of a high reach, studies show that shifting tasks to clients via virtual remote services, particularly for non-complex STI testing and treatment, may be cost-effective (Baraitser *et al.*, 2015; Blake *et al.*, 2015; Wilson *et al.*, 2017). Online services can be an effective and accessible alternative for screening clients for STIs without overburdening established services in some high-risk populations such as young people (Gasmelsid *et al.*, 2021).

Testing guidelines (LCI, n.d.) demand that young heterosexual clients are only tested for chlamydia and gonorrhoea unless specific risk factors are present. This means that no physical examination is needed for the majority of these clients. For this group, home-based test packages in combination with video consultation (VC) can offer an interesting and attractive alternative for the F2F consultations at the clinics. In the Netherlands, 99.2% of young people aged 12–25 years have home internet access (CBS, n.d.; Eurostat, 2021). A smartphone is used by 92% of the Dutch population aged 12 years or older (CBS, n.d.).

Although some studies in other medical fields show that VCs are equivalent to F2F consultations in the terms of patient’s satisfaction and perceived quality of care (Kruse *et al.*, 2017, Barsom *et al.*, 2020), other studies question whether young people will accept VCs in the context of their sexual health. When given a choice, they might prefer telephone consultation (Garrett, C, e.a., 2012). Major doubts that arise in a VC related to privacy and security issues (e.g., that the consultations are recorded and saved) (Garrett *et al.*, 2011).

Given the potential added value of online sexual health care services, the Dutch SHCs wanted to gain insight into whether VCs:

(1) are accepted and appreciated by young clients and nurses;
(2) can offer sufficient quality of care, and—if so—for which SHC services and for which target groups; and
(3) are differently evaluated compared to F2F consultations.

## MATERIALS AND METHODS

A mixed-methods study was conducted to reach this aim. Telephone interviews with clients and focus group interviews with nurses were conducted to provide more in-depth insights into the results of a quantitative evaluation by the means of online questionnaires. The online questionnaires for clients and nurses, the interview guides, the codes used during analyses of qualitative data, a figure of the research process, a table with demographic characteristics of the clients and a table with types of help requests are available at: https://osf.io/mq9rt/?view_only=1ab04676e28547ff95925b3a70349d4c

### Study design

This study was performed among young clients (aged 15–25 year) of nine SHCs in the Netherlands between December 2017 and January 2019. Clients who contacted SHCs with a consultation request had to engage in the standard triage procedure first. The triage was based on age, sexual history and risk behaviour. Only clients with a low urgency and who met the access criteria of an SHC were asked if they would prefer a VC or an F2F consultation. Exclusion criteria for a VC were indications of multiple problems, victim of sexual violence or the need for physical examination at the clinic. In principle, all the consultations were one-time contact consultations.

#### Sample selection VC clients

At the end of each consultation, all the VC clients were asked by the nurse to fill in an online evaluation questionnaire. Those who were willing received the questionnaire directly via e-mail. The questionnaire ended with an invitation to participate in a telephone interview. Clients who were willing to do so submitted their telephone number. Participants in the interviews were offered an incentive of €25. The sample for the interviews was made with the aim of recruiting a heterogeneous sample in the terms of sex, age, educational level, sexual diversity and ethnic background.

#### Sample selection F2F clients

During a period of two months, all the nurses who also conducted VCs were asked to also evaluate their F2F consultations at their clinics. At the end of an F2F consultation, nurses asked their young client if they would like to participate in this study. Those who agreed made use of a tablet of the SHC with a direct link to the online questionnaire which was the same as the one for VC clients.

#### Sample selection VC nurses

The SHCs themselves determined how many VCs they scheduled per week. The three SHCs that first started with the VCs scheduled about 8–12 VCs per week, SHCs that joined the pilot later scheduled a lower number per week.

The VCs were performed by nurses who had previous experience with F2F consultations at an SHC and volunteered to do the VCs. Most of them were also experienced in providing counselling by chat. All the nurses received a training course about the software and points of attention during a VC compared to an F2F consultation prior to the study (e.g., taking into account less non-verbal communication and possible technical failures during VCs). Two SHCs started with VCs, with other SHCs following one-by-one after 6 months.

Nurses were asked to fill in an online questionnaire directly after each VC. Only nurses who started VCs in the first phase of the pilot, and had gained extensive experience with them, participated in a follow-up qualitative evaluation consisting of two focus group interviews.

During a period of 2 months, nurses who asked their clients to evaluate the F2F consultation were also asked to evaluate the consultation themselves directly afterwards using the same online questionnaire as for VCs.

#### Protocol VC

Clients who preferred a VC received a confirmation email containing a personal link with the date and time of the VC appointment, privacy regulations, (technical) instructions, preferred browsers and other points of attention for the VC. One day before the appointment, clients received a reminder by email. At the appointed time of the consultation, the client was directed through a personal link into a virtual waiting room. Before admission to the waiting room, it was indicated that the SHC does not store or share images of the VC with third parties. In this waiting room, a video was shown explaining the procedure of the VC.

### VC equipment

Stand-alone software was used to enable secure VC connection (Webcamconsult BV, Bergen op Zoom, the Netherlands). The software makes it possible to perform VCs on a smartphone, tablet, laptop or desktop computer. Appointments for VCs were enabled to be scheduled using only a first name or nickname and e-mail address. Communication by chat and the exchange of digital files was integrated into the virtual consultation room. VCs were not recorded. All the VC nurses had previously received instructions on how best to organise their workplace for conducting VCs. These instructions included lighting, background, noise reduction and dress codes.

### Data collection

#### Quantitative evaluation by clients

The 14-item online questionnaire consisted of three parts. The first part included questions about the type of consultation that was to be evaluated (VC or F2F), personal preference for one of the consultation types, and in the case of a VC the device used (phone, tablet, laptop or pc). An open text field allowed clients to further elaborate on their preference for the type of consultation. The second part of the questionnaire consisted of questions about appreciation of the consultation. The answers were collected using a 7-point Likert scale ranging from very difficult or bad to very easy or good and a report grade (scale 1–10). The third part collected information about the client’s background such as age, gender, educational level, sexual preference and ethnic background. The online questionnaire concluded with an open space for any comments about the consultation.

#### Qualitative evaluation by clients

The clients who indicated in the online questionnaire that they were willing to participate in the follow-up qualitative evaluation were approached within a week of the VC. An interviewer (from a pool of three) conducted the individual interviews by phone, which lasted approximately 20 minutes. The interviewer asked the client verbally for consent to participate in the study and indicated that participation was anonymous. A semi-structured interview guide was used containing 17 open-ended questions related to the assessment of the VC and factors contributing to this assessment. The interviews were recorded using a digital voice recorder (VN-731PC, Olympus).

#### Quantitative evaluation by nurses

The online questionnaire completed by the nurses consisted of 14 items and was divided into three parts. Part one included questions about the type of consultation (VC or F2F), kind of help request(s) and the perceived complexity of the request. The second part consisted of questions concerning the appreciation of the quality of the consultation. The answers were collected using a 7-point Likert scale ranging from totally disagree or very bad to totally agree or very good. The third part collected information about the client’s background such as age, gender, educational level, sexual preference and ethnic background.

#### Qualitative evaluation by the nurses

The two focus group interviews were organised at the two different SHC locations: Groningen (13 March 2018) and Heerlen (9 April 2018). Two of the interviewers who conducted the interviews with the clients were present, with one acting as moderator of the discussions. A semi-structured interview guide consisting of 16 open-ended questions was used. Before the start of the discussion the moderator asked the nurses verbally for consent for participation in the study and indicated that participation was anonymous. The focus group interviews were recorded using a voice recorder (VN-731PC, Olympus).

### Data processing and analysis

#### Online questionnaires

IBM SPSS V.25 was used to provide descriptive statistics per type of consultation and sub-groups of clients based on gender, educational level and age. Independent samples *t*-tests were conducted to indicate the level of significance of differences between sub-groups. Due to an insufficient number of participants per sub-group, it was not possible to provide descriptive statistics per sexual orientation and different ethnic backgrounds.

#### Telephone and focus groups interviews

All completed interviews and focus groups were transcribed and names of participants were deleted. OTranscribe and the software program ‘Transcriptions’ were used to transcribe the interviews. All the transcripts were checked for possible mistakes. QSR NVivo (V.11, QRS International) was used to analyse the qualitative data.

A thematic analysis was used. The themes were deductively determined based on general pre-specified assessment criteria for consultations. At the same time, we left open the possibility to inductively add codes based on data. The data was coded by one of the researchers who conducted a part of the interviews with the young clients and who was also present at the two focus group sessions with the nurses. The codes were discussed and reviewed by the all the interviewers and the moderator of the focus groups.

## RESULTS

In total 474 VCs were initiated in the study period. Based on analysis of the reactions in the open spaces for remarks in the questionnaires, it can be concluded that technical problems occurred on a regular basis. Of the 118 remarks made by clients, 39 were related to technical problems. The nurses made 175 comments, 105 of which were related to a technical malfunction. The most frequently mentioned problems were related to a poor internet connection, malfunction of the webcam or the usage of a non-compatible browser. In most cases, the technical problems were resolved or the VCs were resumed with only an audio connection.

### Demographic characteristics of clients

Young clients evaluated 333 VCs (69%) of the total 474 VCs initiated. The majority of the participants—both for VCs and F2F consultations—were female, had attained a higher educational level, had a native Dutch background and were heterosexual. This is basically in line with general characteristics of young low urgent clients of regular consultations at SHCs (Staritsky, 2020). People with a lower level of education or with a non-Dutch origin were not sufficiently reached by the regular consultations of the SHCs. On average, 60% of the clients with SHC have a high educational level, 30% an intermediate level and only 10% have low education level. Persons with a low level of education are more likely to request a test at a GP (Heijne *et al.*, 2019). Young MSM were only asked to a very limited extent whether they wanted to participate in a VC because they were often categorised as high urgency clients. Most clients of the VCs (70%) used a laptop or desktop computer, while a quarter (25%) used their smartphone. Only 1% used a tablet.

### Clients’ evaluation of the VCs

Clients’ responses to the online questionnaire are shown in Table 1. Both VCs and F2F consultations were very positively assessed. For both groups, communication with the nurse was comfortable and the quality of the contact was evaluated as high. Both types of consultation offered good opportunities to ask any questions attendees might have had. Satisfaction with the advice given by the nurses was high for both consultation types. In line with the positive assessment of these different quality aspects, the summary grade for consultations was very high, with an average report grade of 8.7 for the VCs and an 8.9 for the F2F consultations.

**Table 1:** Evaluation of consultations by clients and sub-groups

Items 1–4: 7-point Likert scale Item 5: report grade (scale 1–10), see Data collection section				M (SD)			
				VC (*n*=333)		F2F (n = 100)	
1. How did you feel about talking about sexuality with the SHC nurse?				6.27 (0.84)		6.30 (0.95)	*t*(431) = 0.27, *p* = .79
2. What did you think of the contact with the SHC nurse?				6.69 (0.55)		6.74 (0.46)	*t*(431) = 0.86, *p* = .39
3. To what extent do you feel that all your questions have been addressed?				6.74 (0.56)		6.85 (0.39)	*t*(431) = 1.70, *p* = .09
4. What did you think of the SHC nurse’s advice?				6.63 (0.63)		6.68 (0.62)	*t*(431) = 0.65, *p* = .52
5. What grade would you give the consultation?				8.71 (0.94)		8.88 (1.00)	*t*(431) = 1.60, *p* = .11
Evaluation of VCs by sub-groups							
		*N*	M (SD)	M (SD)	M (SD)	M (SD)	M (SD)
			Item 1	Item 2	Item 3	Item 4	Item 5
Gender (*n* = 332)	Female	263	6.23 (0.85)	6.71 (0.54)	6.76 (0.54)	6.67 (0.61)	8.69 (0.95)
	Male	69	6.45 (0.76)	6.61 (0.60)	6.71 (0.64)	6.51 (0.72)	8.77 (0.94)
			*t*(df) = 1.96 *p* = 0.05	*t*(df) = 1.32 *p* = 0.19	*t*(df) = 0.66 *p* = 0.51	*t*(df) = 1.90 *p* = 0.06	*t*(df) = 0.63 *p* = 0.53
Education (*n* = 332)	Low/ Intermediate*	51	6.18 (0.91)	6.63 (0.63)	6.73 (0.67)	6.65 (0.56)	8.63 (1.08)
	High	281	6.29 (0.82)	6.70 (0.54)	6.75 (0.54)	6.63 (0.65)	8.72 (0.92)
			*t*(df) = 0.88 *p* = 0.38	*t*(df) = 0.83 *p* = 0.41	*t*(df) = 0.30 *p* = 0.77	*t*(df) = 0.18 *p* = 0.86	*t*(df) = 0.64 *p* = 0.53
Age (years) (*n* = 333)	≤ 18	30	6.10 (0.92)	6.43 (0.73)	6.50 (0.94)	6.33 (0.61)	8.60 (0.81)
	19-24	303	6.29 (0.83)	6.71 (0.53)	6.77 (0.51)	6.66 (0.63)	8.72 (0.96)
			*t*(df) = 1.20 *p* = 0.24	*t*(df) = 2.67 *p* = 0.008	*t*(df) = 2.55 *p* = 0.01	*t*(df) = 2.75 *p* = 0.006	*t*(df) = 0.64 *p* = 0.52

Table 1 also summarizes the results of the evaluation of VCs by demographic characteristics of the clients. The differences in assessment of the VCs between men and women, low/intermediate and higher educated and younger and older clients are negligible. Clients aged between 19 and 24 years are slightly more positive about the contact with the SHC nurse.

## RESULTS OF THE TELEPHONE INTERVIEWS WITH CLIENTS

### Perceived advantages by clients

A total of 16 individual telephone interviews were conducted with clients of VCs to guide interpretation of the results of the quantitative evaluation: 11 women, 4 men and 1 transgender were interviewed. The majority of the interviewees were higher educated (63%), heterosexual (75%), aged between 20 and 24 years (75%) and of the native Dutch origin (81%).

The positive assessment in the quantitative evaluation is confirmed by the results of telephone interviews among clients of VCs. The most important perceived advantages were time saving, ease and feelings of comfort and safety. This was because the clients did not have to travel to the SHC and could attend from their home. Additionally, if waiting times for a VC were shorter than those for an F2F consultation, this was mentioned as a very important advantage by those involved. For many young clients, it is easier to use a computer at home instead of going to the SHC. They mentioned feeling more anonymous and safer: not having to worry about encountering an acquaintance at the SHC.

I liked it. And precisely because you are at home, enjoying a cup of tea in your own place, I really liked that. Instead of having to go there and then yes, one on one, that might be a bit scarier. You feel a little more self-confidence from your own home.

Important aspects contributing to the positive evaluation of the contact with the SHC nurse were the pleasant and relaxed atmosphere during consultations, the genuine interest of the nurse and the openness during the entire consultation. Nevertheless, interviewees differed slightly in their opinion about whether the VC would offer the same depth of the questions addressed compared with an F2F consultation.

Other interviewees stated that they experienced no difference between a VC and an F2F consultation:

I actually found it easy, just like a normal conversation at the SHC. Not much changed for me, it was now just online and via a webcam. But in principle I had the idea as if I was just sitting in that room at the SHC.

Participants were asked about their preference for an online consultation with or without video. The majority preferred a combination of video and audio, as it enabled them to see who they were talking to. They indicated that—compared to a telephone consultation—it felt safer and that it was good to see non-verbal reactions:

Yes, yes that gives just a little extra contact if you can see each other. And that someone shows an attitude of: hey you, I’m listening to you and understand you.

A number of interviewees, who only had an audio connection due to technical interference, did not find this annoying but missed the personal aspects such as eye contact and non-verbal communication. In line with these results, the interviewees did not recommend using only chat for an online consultation. They considered that it would be too difficult to express themselves or to feel emotions in their contact with the nurse, for example, a change in the tone of voice.

### Perceived disadvantages by clients

While more benefits were mentioned in the interviews, some drawbacks were also addressed. Most concerned technical issues such as a bad internet connection. Another area for improvement was the instructions and explanation of the process of home-based testing.

Most of the interviewees would prefer a VC in the future if they needed a consultation at the SHC. If there was no difference in waiting time between an F2F consultation and a VC, the preference for a VC was less clear. For more sensitive and personal issues, interviewees were inclined to prefer an F2F consultation. That would also be their advice to other young people:

I think it’s actually very personal. Because some people find it easier to talk about a sensitive topic than others. Some find it easier face to face, for others it is better to do it from home in a safe environment.

### Nurses’ evaluation of the VCs

In total, nurses of nine SHCs evaluated 542 consultations: 422 VCs (89% of the VCs initiated in total) and 120 F2F consultations. Almost all consultations were STI consultations. Due to exclusion criteria of VCs, the help requests during the F2F consultations were relatively more complex and concerned relatively more clients with multiple issues.

Table 2 shows an overview of the average scores of nurses on the evaluation questions. The contact in a general sense with the client, the extent to which there was a possibility to continue asking questions and the appropriateness of the type of consultation with regard to the request for help were all assessed positively by the nurses for both types of consultation. On all items except for item 3 (estimation of the complexity of the primary request), the average scores were slightly higher for the VCs. In accordance with the exclusion criteria of VCs, F2F consultations were assessed as being more complex.

**Table 2:** Evaluation of consultations by nurses

Items 1–4: Likert scale 1–7, see Data collection section				M (SD)		
				VC (*n* = 422)	F2Fc (*n* = 120)	
1.How did you find the contact with the client in a general sense?				6.09 (1.07)	5.82 (1.29)	*t*(df)=2.36, *p* = 0.02
2.I managed to keep asking questions during the consultation				5.89 (1.24)	5.87 (1.24)	*t*(df) = 0.21, *p* = 0.84
3.How do you estimate the complexity of the primary request for help in this consultation?				1.93 (1.28)	2.72 (1.60)	*t*(df) = 5.62, *p <* 0.001
4.The consultation method (VC or F2Fc) was suitable for the client’s request for help.				6.16 (1.30)	5.84 (1.67)	*t*(df) = 2.23, *p* = 0.03
Evaluation of consultations by nurses according to client sub-groups						
		N	M (SD)	M (SD)	M (SD)	M (SD)
			Item 1	Item 2	Item 3	Item 4
Gender (*n* = 421)	Female	320	6.08 (1.07)	5.92 (1.22)	2.00 (1.30)	6.07 (1.36)
	Male	101	6.10 (1.05)	5.81 (1.32)	1.69 (1.17)	6.45 (1.03)
			*t*(df) = 0.12 *p* = 0.90	*t*(df) = 0.73 *p* = 0.47	*t*(df) = 2.10 *p* = 0.04	*t*(df) = 2.54 *p* = 0.01
Education (*n* = 409)	Low/intermediate	77	5.91 (1.00)	5.60 (1.28)	2.18 (1.36)	5.87 (1.61)
	High	332	6.17 (1.07)	6.00 (1.20)	1.85 (1.24)	6.26 (1.20)
			*t*(df) = 1.92 *p* = 0.06	*t*(df) = 2.63 *p* = 0.009	*t*(df) = 2.08 *p* = 0.04	*t*(df) = 2.37 *p* = 0.02
Age (years) (*n* = 422)	≤ 18	35	6.14 (1.12)	6.11 (1.13)	1.83 (1.29)	6.20 (1.37)
	18-24	387	6.09 (1.06)	5.87 (1.25)	1.94 (1.28)	6.16 (1.29)
			*t*(df) = 0.31 *p* = 0.76	*t*(df) = 1.10 *p* = 0.27	*t*(df) = 0.49 *p* = 0.63	*t*(df) = 0.19 *p* = 0.85

Table 2 also summarises the results of the evaluation of the VCs by demographic characteristics of the clients. The differences are minor. The possibility to continue asking questions is evaluated a little more positively among higher educated clients. The nurses assess the appropriateness as a little higher for men and for higher educated clients.

## RESULTS OF THE FOCUS GROUP DISCUSSIONS WITH NURSES

### Perceived advantages by nurses

In line with the results of the quantitative evaluation, the nurses who participated in the two focus groups discussions were positive about the VCs. Some of them acknowledged that they thought the VCs went better than they initially thought. The accessibility of the VCs was mentioned as a positive element and they reported the VCs as being of added value to F2F consultations. The nurses felt that they were getting to the point faster during a VC, so VCs took less time compared to F2F consultations:

You do not have all kinds of actions around it, letting someone in, going to the toilet, taking off your coat, hanging it on the coat rack, which means that a lot of interference disappears.

### Perceived disadvantages by nurses

However, other activities surrounding VCs, like organising the evaluation, completing the medical file and sending the home-testing-packages took extra time. They realised that some of these activities were related to the pilot design of the project.

Compared to an F2F consultation, the nurses felt that a VC had less impact, especially due to the loss of some non-verbal communication, as normally only a client’s face is visible during a VC. Furthermore, less eye contact was perceived as a disadvantage of a VC. But for a standard STI consultation for clients without any additional problems, VCs were considered as a possible good alternative.

Nurses regard a well-functioning technology as the most important prerequisite for a successful VC. Technical problems cause delays and weaken contact with the client. Most of these problems occurred on the client side:

Because the client was unable to log in via the webcam, we had contact by telephone. Because she also appeared to have problems with shame and unacceptable behavior, I would have liked to have seen her. Whether that would have been face to face or via the webcam, I don’t think that makes much difference.

## DISCUSSION

### Client perspective

The young clients of SHCs appreciate and accept a VC on a similar level as an F2F consultation, regardless of their age, educational level or gender. This is in line with the results of recent studies within primary and secondary care (Tates *et al.*, 2017; Greenhalgh *et al.*, 2018, 2020; Donaghy *et al.*, 2019; Hammersley *et al.*, 2019; Barsom *et al.*, 2020). An important difference with the present study is the relationship between the client and the healthcare provider. In this study, the VCs were used for one time contact, while the VCs in the cited studies are follow-up consultations between clients and clinicians who have already established a relationship. An existing patient–clinician relationship in combination with a condition that is already diagnosed, are regarded by both patients and clinicians as important prerequisites for a successful experience of remote consulting (Greenhalgh *et al.*, 2018; Donaghy *et al.*, 2019; Hammersley *et al.*, 2019). The results of this study confirm that a VC can also be used for one-time consultations.

Another concern emerging from these recent studies is the suitability of VCs for highly personal topics such as sexual health. The present study shows that this concern is hardly an issue for most young clients of SHCs, provided that the VCs are offered by nurses who have experience in discussing sexual health. This is confirmed by a review on barriers of SRH services (Bender and Fulbright, 2013) in which an important advantage of VCs for certain clients lies in the fact that consultations from home without being seen by others, increases the feeling of safety and privacy. This is especially true for clients who are not comfortable with the risk of running into acquaintances at the SHC. A VC enables them to bypass this risk. On the other hand, clients may envisage privacy issues while conducting a consultation from home, such as disturbances by parents or housemates. It is important that clients are offered a choice for the type of consultation that suits them best.

Travelling to the clinic is a barrier frequently mentioned in international research by young clients of SRH services (Bender and Fulbright, 2013). A recent Dutch study (Twisk *et al.*, 2021) has also shown that SHC visits of clients living further away from the SHC are less frequent compared to those living closer. The present study provides evidence that SHCs adding VCs to their services will render services more attractive—and potentially increase accessibility—for all the clients.

The combination of video and audio plays an important role in the quality assessment. In this study, compared to a telephone consultation, a VC was judged to be more personal and safer because of the possibility of making eye contact and seeing non-verbal reactions. This is in line with findings of other recent studies (Donaghy *et al.*, 2019; Hammersley *et al.*, 2019). It may be argued that these benefits may not be seen by young people who have never had a personal experience with a VC. An Australian qualitative study (Garrett, C, e.a., 2012) concluded that VCs for sexual health may not yet be acceptable to young people due to a dominant preference for telephone consultation. The main reasons for this preference include not owning a webcam, convenience, familiarity with telephone and finding video too confronting. In recent years, however, both the availability and usage of various devices such as smartphones, laptops and webcams by young people have strongly increased (CBS, n.d.). Because of COVID-19, their experience with video conferencing may have greatly increased. Clients who already used Skype or FaceTime socially and/or at work feel more comfortable with VCs (Donaghy *et al.*, 2019). Experience with video calling may have a greater influence on the assessment of a VC than the education level of a client. Patients with experience using video calling in daily life choose a VC over F2F consultation more often (Barsom *et al.*, 2021).

Despite the great accessibility of home-based internet and smartphones, especially in the Netherlands, it is very important to take into account that not all groups use them or choose to use them for health purposes. In the Netherlands, health-seeking behaviour on the internet depends on educational background. High-level educated people look for health information on the internet more often (84%) compared with those with intermediate (76%) and lower education levels (57%)(CBS, 2021). Also, digital skills vary, depending on educational and cognitive level (Office for national statistics, n.d.; Pharos, 2021). Before offering VC to a client, it would be advisable to perform a quick scan to test their digital skills. VCs should be offered as an additional service, not as a replacement for F2F consultations for everybody.

In those cases where travel distance plays a limited role, this study indicates that young people do not have a strong preference when offered either a VC or an F2F consultation. The waiting time for consultation strongly determines their preference. Therefore, should SHCs choose to structurally implement VCs, they can make VCs more attractive to young people by reducing waiting times.

### Health providers perspective

According to the nurses, VCs can be an attractive addition to the services of SHCs. They assess the quality of VCs as being similar to F2F consultations for standard—not complex—consultations. This is consistent with results of other studies on the implementation of VCs within primary and secondary health care. (Greenhalgh *et al.*, 2018; Donaghy *et al.*, 2019; Hammersley *et al.*, 2019). Various nurses indicated that VCs actually went better than they had anticipated beforehand. This underlines the importance of considering possible doubts among the professionals involved about the quality of VCs when implementing them. For example, by having the colleagues who have gained experience with VCs act as ambassadors. They can share their experiences and lessons learned during presentations and peer meetings. In the earlier work, computer illiteracy was discussed as a possible barrier among healthcare providers (Barsom *et al.*, 2020), which demands for access to appropriate training (Hanna *et al.*, 2012). It is therefore wise to organise VC training courses and test consultations for nurses who have doubts about their computer skills.

The results of qualitative evaluation of VCs by nurses raise the question of whether VCs are more suitable for clients with a high level of education. Based on the similar assessments by lower/intermediate and higher educated clients and the limited difference in assessment by nurses, our study does not provide evidence for a recommendation to exclude lower/intermediate educated clients from a VC. But it does emphasise the importance of clear, simple and preferably pretested instructions about the VC procedures and technology.

According to the nurses, most technology problems such as no sound or no video function occurred on the client side. A well-functioning technology is regarded as the most important prerequisite for the successful use of VCs as they can disrupt the consultation process. Although technical problems are more commonly reported with VCs than with telephone consultations (Hammersley *et al.*, 2019), in many cases, they could be resolved in a relatively simple way, as is also confirmed in another study (Greenhalgh, T. 2018). Nevertheless, good ICT support remains of great importance when implementing VCs.

### Limitations of this study

A limitation of this study is that participants are mostly higher educated, heterosexual and of native Dutch origin. Participants with a non-heterosexual sexual orientation (homosexual, lesbian or transgender) and/or with a migrant background are under-represented. Therefore, the results of this study cannot be generalised to these specific client groups. Although we asked the participating SHCs to explicitly invite lower educated people to use the VCs, the relatively limited number who reported to the SHCs unfortunately showed less interest in the possibility of a VC or were categorised as ‘high-urgency clients’.

Another limitation of this study concerns the assignment of clients to VCs. Clients were given the option to choose a between a VC or an F2F consultation. In many cases, the waiting time for a VC was shorter than for an F2F consultation. Unfortunately, the waiting time was not systematically registered per consultation for study purposes. We cannot, therefore, identify from this study which preferences clients have prior to the consultation solely based on their expectations regarding a VC.

Given the nurses’ assessment that the suitability of a VC depends on the complexity of a help request, an accurate assessment of the complexity of the two consultation types compared would have been desirable. This would provide more insight into the possible influence of this complexity on the assessment of consultations. Future research should focus on this relationship and explore the additional value of VCs in follow-up consultations: sharing test results and treatment consultations in the case of positive STI test results.

To promote participation and completion of the study, the number of items in the quantitative evaluation were limited to the main issues. Questions about specific characteristics of VCs like a lack of direct eye contact or less nonverbal communication were not included. Neither were questions about the occurrence of any technical problems included. Insight into the influence of these factors on the assessment of VCs was obtained by performing additional qualitative research among both clients and nurses.

## CONCLUSION

The VCs are accepted and appreciated by both young clients and nurses of SHCs. Their quality is sufficient for standard one-time contact STI consultations that do not require a physical examination. For these types of consultations, VCs and F2F consultations are evaluated similar. The advantages of VCs can contribute to reaching target groups that make less use of the current services of SHCs, i.e., clients who live further away from an SHC and clients who do not wish to be seen at an SHC, or who for other reasons prefer a consultation from their homes.
